# Considerations and Influencing Parameters in EDS Microanalysis of Biogenic Hydroxyapatite

**DOI:** 10.3390/jfb11040082

**Published:** 2020-11-15

**Authors:** Florin Miculescu, Cristina Luță, Andreea Elena Constantinescu, Andreea Maidaniuc, Aura-Cătălina Mocanu, Marian Miculescu, Ștefan Ioan Voicu, Lucian Toma Ciocan

**Affiliations:** 1 Faculty of Materials Science and Engineering, University Politehnica of Bucharest, 313 Splaiul Independentei, 011061 Bucharest, Romania; cristina.luta27@yahoo.com (C.L.); andreeaelena01c@gmail.com (A.E.C.); mcn_aura@hotmail.com (A.-C.M.); marian.miculescu@gmail.com (M.M.); 2 S.C. Nuclear NDT Research & Services S.R.L, 104 Berceni St., Central Laboratory Building, 041919 Bucharest, Romania; andreea.maidaniuc@gmail.com; 3 Department of Analytical Chemistry and Environmental Engineering, University Politehnica of Bucharest, 011061 Bucharest, Romania; svoicu@gmail.com; 4 Department of Prosthetics Technology and Dental Materials, University of Medicine and Pharmacy, 020022 Bucharest, Romania; tciocan@yahoo.com

**Keywords:** calcium phosphates biomaterials, EDS results influencing parameters, calcium phosphorus ratio

## Abstract

Calcium phosphates (CPs) used as biomaterials have been intensively studied in recent years. In most studies, the determination of the chemical composition is mandatory. Due to the versatility and possibilities of performing qualitative and quantitative compositional analyses, energy dispersive spectrometry (EDS) is a widely used technique in this regard. The range of calcium phosphates is very diverse, the first method of approximating the type of compound being EDS microanalysis, by assessing the atomic Ca/P ratio. The value of this ratio can be influenced by several factors correlated with instrumental parameters and analysed samples. This article highlights the influence of the electron beam acceleration voltage (1 kV–30 kV) and of the particle size of calcium phosphate powders on the EDS analysis results. The characterised powders were obtained from bovine bones heat-treated at 1200 °C for 2 h, which have been ground and granulometrically sorted by mechanical vibration. The granulometric sorting generated three types of samples, with particle sizes < 20 μm, < 40 μm and < 100 μm, respectively. These were morphologically and dimensionally analysed by scanning electron microscopy (SEM) and compositionally by EDS, after the spectrometer was calibrated with a standard reference material (SRM) from NIST (National Institute of Standards and Technology). The results showed that the adjusting of acceleration voltage and of the powder particle size significantly influences the spectrum profile and the results of EDS analyses, which can lead to an erroneous primary identification of the analysed calcium phosphate type.

## 1. Introduction

Microanalytical methods, coupled to those of electron microscopy, allow for the identification and quantification of the chemical elements (usually from boron to uranium) that are present on the surface or within samples, depending on the sample preparation method. X-ray microanalysis techniques (energy dispersed spectrometry (EDS), or wavelength dispersed spectrometry (WDS)) use the X-rays generated by a sample bombarded with an accelerated electron beam [1,2]. Both techniques enable the obtaining of emission spectra in the range of X-rays, in which the maxima correspond to the energies or wavelengths characteristic of the chemical elements and allow their rapid analysis (10 s) [1,3,4]. Due to versatility and instruments price, of the two, the most popular and used is the EDS method.

By complementarity with the scanning electron microscopy (SEM) analysis [1,5,6,7], the morphological characteristics can be correlated with those of elemental chemical composition of some regions with cubic micrometres order [8,9,10]. Usually, the analytical resolution depends on the incident beam energy, the atomic mass, the atomic number and the density of the sample, respectively [11], with the more difficult being the analysis of light elements, such as those present in the composition of calcium phosphates. Irradiation of a sample with an electron beam causes a transfer of energy to the sample, which can induce its degradation. Therefore, a high value of the acceleration voltage leads to an increase in temperature and, at the same time, induces the risk of degradation of sensitive samples (in this study experiments, the acceleration voltage was swept in the range 1–30 kV).

Data collection and EDS analysis is a relatively fast process, due to the full spectrum of energy that can be simultaneously acquired. Through WDS, the spectrum is obtained sequentially, and the entire wavelength range is composed of multiple acquisitions. Although it takes a longer time to obtain a full spectrum, the WDS technique has a much better resolution than EDS [8], but it is more expensive and the results are more difficult to interpret. However, EDS is widely used for elemental analysis of biomaterials [12].

In the biomedical field, elemental EDS analysis is used for many purposes, including the detection of heavy residual elements in tissues and organs [13], the analysis of mineral deposits (stones formed in various organs) and calcified tissues present in the body (Ca, P, Mg) [14] with supporting role in the skeletal system [14,15]. Numerous studies have approached both morphological [16] and compositional [17,18] methods for the analysis of calcium phosphate composition [19,20,21]. In some studies, EDS analysis was used to evaluate the chemical composition of the median aortic calcification [22] and the genesis of Ca and P deposits in atheroma plaques [23].

The range of calcium phosphates (CPs) is very diverse [24], with some of them having wide applicability in the biomedical field [17,19]. The best known and used compounds of this type are hydroxyapatite (HAP), and alpha- and beta-tricalcium phosphates (α-TCPs and β-TCPs) [6,25]. The identification of calcium phosphate compounds can be performed rapidly by EDS microanalysis, by assessing the atomic percentages of Ca/P ratio. The value of this ratio can be influenced by systematic errors generated by several factors correlated with the instrumental parameters (e.g., beam acceleration voltage), and with the analysed samples (particle size). Moreover, the development of the field of electron microscopy by the appearance of SEM desktop instruments involved the reduction of the acceleration voltage of microscopes to 15 kV, in this case, not being possible to achieve an optimal overvoltage ratio for all detectable elements.

The particle size from CPs is important in the biomedical field from several perspectives. When used in implantology, the bioresorption rate can be modulated depending on the type of CPs and the powder particles sizes [19,24,26]. In some cases, it is possible to obtain customised products using additive manufacturing (AM) techniques [2,21]. The characteristics of bioceramic products obtained by these methods can be influenced by both size and shape of the particles [17,25]. In the case of implants obtaining by AM, the particle size contributes, among other things, to the definition of the porosity and to the dimensional accuracy of the obtained products [2,27]. In addition, particle size also has an effect on the biological properties of CPs [28], the kinetics of drug release from transporting particles [29] and, last but not least, on the pastes viscosity (especially in the case of robocasting method) [30]. All these considerations lead to the use of CPs powders with particles from a certain dimensional range, which are also characterised by EDS. This aspect led to the idea of this research, with the article being important for the field approached by highlighting, for the first time, the considerations and parameters that influence the compositional characterisation by EDS microanalysis of CPs type biomaterials, which are usually in powder form, in a certain dimensional range.

## 2. Materials and Methods

### 2.1. Synthesis of Hydroxyapatite Particles

Precursor materials taken from the femoral component of bovine bone tissue were used to obtain CPs powders. The powder was obtained and complexly characterised as previously described in the literature [20,31]. These data show that the used powders are non-stoichiometric hydroxyapatite. Bone samples were heat treated in air at 1200 °C (with a heating rate of 10 °C/min) for 2 h [18,25,32]. After air cooling, the samples were ground in a mill with agate balls and then granulometrically sorted in a sieve with predetermined dimensions. Three-dimensional sorts were obtained, with particles < 20 μm; < 40 μm and < 100 μm, respectively.

### 2.2. Characterisation Methods

The morphology of the CPs powders was studied by scanning electron microscopy (SEM) with a Philips XL 30 ESEM TMP microscope (FEI/Phillips, Hillsboro, OR, USA), equipped with a low vacuum secondary electron detector (SE) and a backscattered electrons detector (BSE) with solid body. The samples were mounted on aluminium stubs coated with double carbon adhesive foil and were directly investigated. In order not to influence the result of the micro-analysis by covering the samples with conductive elements, the samples were analysed without further processing, the equipment allowing the analysis of samples in the gaseous environment (ESEM). A water vapour pressure of 0.7 mBar was used in the sample chamber. The analysis of the chemical composition of the powders was performed by energy dispersive spectrometry (EDS), with an EDAX Sapphire UTW microanalysis system, with a resolution of 128 eV, coupled to the Philips XL 30 ESEM TMP scanning electron microscope. To study the influence of acceleration voltage on the results, it was swept between 1 kV and 30 kV, in 1 kV steps.

The spectrometer was calibrated with a standard reference material (SRM) with NIST (National Institute of Standards and Technology) traceability (Standard Reference Material 2910b), used to evaluate the physical and chemical properties of apatites with biological, geological or synthetic origins. The calcium to phosphorus (Ca/P) molar ratio for SRM 2910b is consistent with the theoretical Ca/P molar ratio of 1.67 for hydroxyapatite and a compositional formula of Ca_10_(PO_4_)_6_(OH)_2_ [33].

The particles’ sizes were determined my SEM micrographs processing via ImageJ 1.50 software (Bethesda, MD, United States).

For each particle size range of CPs powders, the Ca/P ratio was calculated taking into account the overvoltage ratio. Equation (1) was used for this purpose:

U = E_0_/E_crit,_ (1)

where U is the acceleration voltage, E_0_ is the energy of the electron beam and E_crit_ is the critical excitation energy of the X radiation. Each sample was analysed 5 times, in randomly selected areas, and the results were expressed as arithmetic mean ± standard deviation.

## 3. Results and Discussions

### 3.1. Morphological Analysis of Calcium Phosphate Powders by SEM Analysis

**Figure 1:**
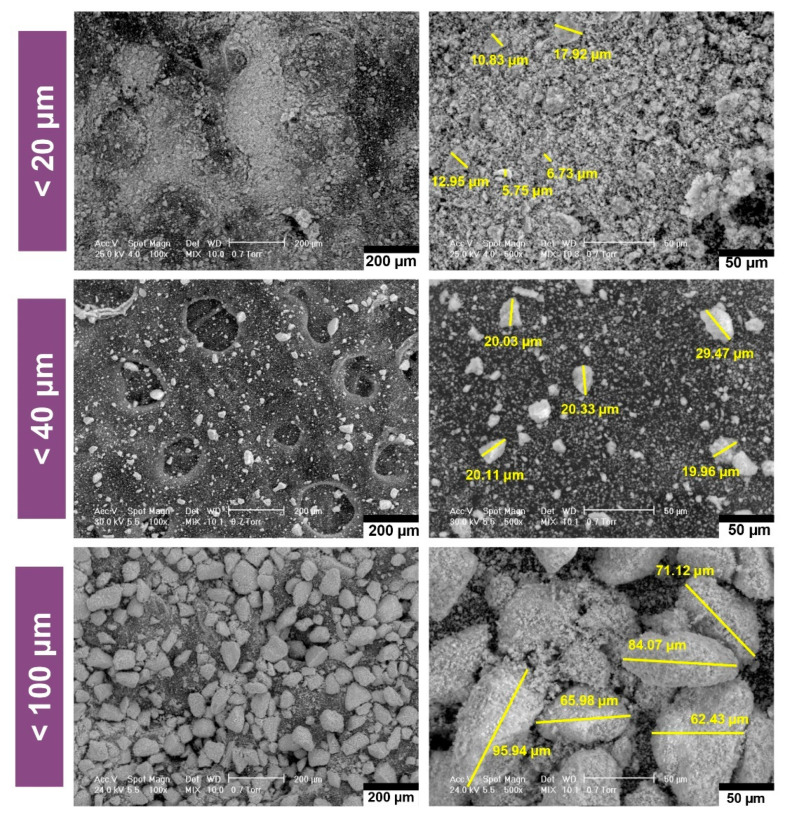
Morphological and dimensional characterisation of calcium phosphates (CPs) particles, sorted by sizes.

In order to obtain relevant results in the field of CPs powders microanalysis, three sorts with very different sizes were chosen, the particles being < 20 μm, < 40 μm and < 100 μm (see Figure 1) respectively. The powders were obtained by particle size sorting by vibration in the sieve.

All the investigated dimensional ranges showed particles with complex geometries, without agglomerations. The analysis of the morphology of the CPs powders revealed both faceted and rounded edges particles, with elongated, irregular shapes and rough surfaces [25]. The maximum particle sizes are in accordance with the mesh sizes of the particle sorting sieves. A uniform dimensional distribution is observed in the case of the sorts with the smallest and largest size, in the case of powder < 40 μm, a mixture from a wider dimensional range being observed.

### 3.2. Influence of Acceleration Voltage on EDS Results

**Figure 2:**
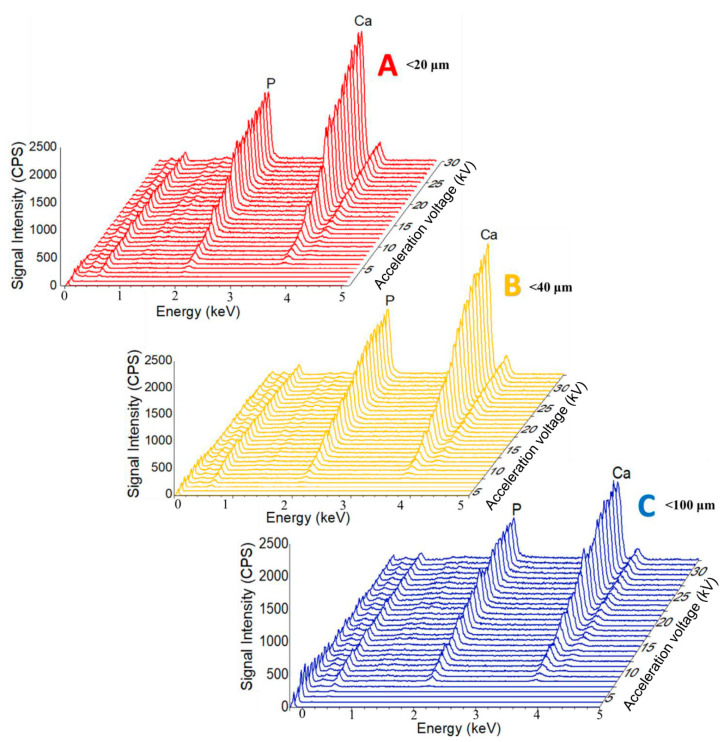
Comparative representation of the variation of the shape and amplitude of the X-ray emission profiles as a function of the acceleration voltage: (**A**) <20 μm; (**B**) <40 μm și (**C**) <100 μm.

Figure 2 compares the EDS spectra corresponding to the analysis of CPs particles from the particle size ranges shown in Figure 1. Working parameters (equal electron shell size, 1200 counts per second, 50 life seconds acquisition time, acceleration voltage range between 1 and 30 kV) used to obtain the EDS results were selected taking into account the capacity of the Si (Li) detector in the EDS system, so as not to impair the quality of the results.

The uncertainty associated with these errors is difficult to determine, but is estimated at about 1% of the measured concentration, varying depending on the analysed chemical element. The X-ray photons having higher energies than the incident energy of electrons that cannot be excited [4]. Therefore, the compositional information is lost at acceleration voltages < 5 kV [34].

From the compositional point of view, the main identified elements and being characteristic of the analysed powder are Ca and P, according to the results of similar studies [6,25,31]. In the case of the EDS method, the analytical information comes from a depth that is generally characteristic for the analysis of thin films (1–10 µm) [25,35]. Thus, the particle size influences both qualitatively and quantitatively the Ca and P ratio. As the acceleration voltage increases (from 1 kV to 30 kV), the increase in signal strength corresponding to the Ca and P elements appears as quasi-linear (up to 15 kV) regardless of the CPs particle size. In the range of 15–30 kV, a variation in the shape of saw teeth is observed for the particle sizes with minimum and maximum dimensions, and uniform in the median case.

The results published in the literature showed that for an energy with a primary beam of 20 kV, the scattering depth of the backscattered electrons in the sample can reach about 1 mm [34]. These statements are mainly based on the fact that the critical energy required to detect characteristic X-rays for the particle size range <100 μm Ca Kα is 3.69 keV, and for P Kα is 2013 keV. EDS microanalysis can also be performed at low voltage, and both the electron domain and the interaction volume are significantly reduced [36]. Thus, both lateral and depth resolution are improved, and absorption losses are reduced for emerging radiation. This phenomenon has been observed by other authors [37]. Notable papers have been reported by Wu et al. [37], who used energy dispersive spectroscopy (EDS) coupled with scanning electron microscopy (SEM) to assess the influence of acceleration voltage on quantitative results.

**Figure 3:**
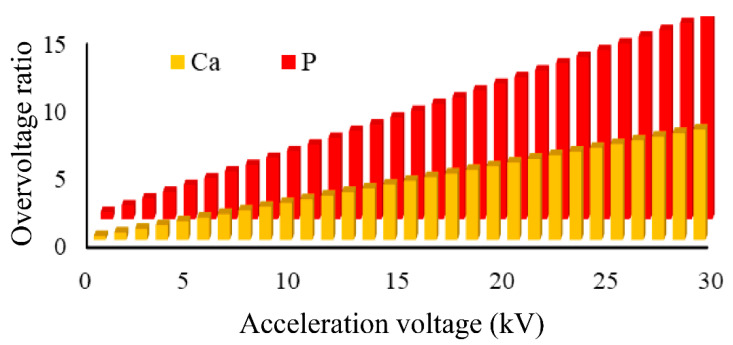
Overvoltage ratio for Ca and P at voltages of 1–30 kV.

From our study, the energy required to excite the CPs characteristic elements was evaluated by means of the overvoltage ratio (Figure 3). The results obtained for the overvoltage ratio corresponding to the Ca and P elements at acceleration voltages in the range 1–30 kV are presented in Figure 3.

The results revealed a significant increase in signal intensity corresponding to Ca and P elements, while increasing the applied voltage. According to the literature [1,25], for sufficient qualitative and quantitative generation of X-rays, the recommended overvoltage ratio should be at least 2, and the optimal recorded ratio should be 2.7 [38].

As the acceleration voltage was reduced to minimise the effects of particle size, the overvoltages for the various characteristic X-ray lines decreased. In many cases, this involves choosing a characteristic line for which the overvoltage is less than the recommended value. The lower the overvoltage, the lower the X-ray intensity than expected for all elements, also stated in other studies [39].

### 3.3. Influence of the Particle Size on EDS Results

The EDS results obtained for ceramic powders with different particle sizes are presented in Table 1. These revealed the presence of Ca and P elements as major components. The Ca/P ratio calculated directly from the EDS results varies between 1.71–1.83, values relatively close to the theoretical value (1.67), characteristic to the stoichiometric hydroxyapatite [25,26].

The corresponding results for Ca and P show an upward trend of the Ca/P ratio concentration, simultaneously with the increase of the analysis depth. The standard deviation (calculated based on five measurements performed under repeatable conditions) are represented in Table 1. The lowest value is recorded for the powder < 40 µm.

**Figure 4:**
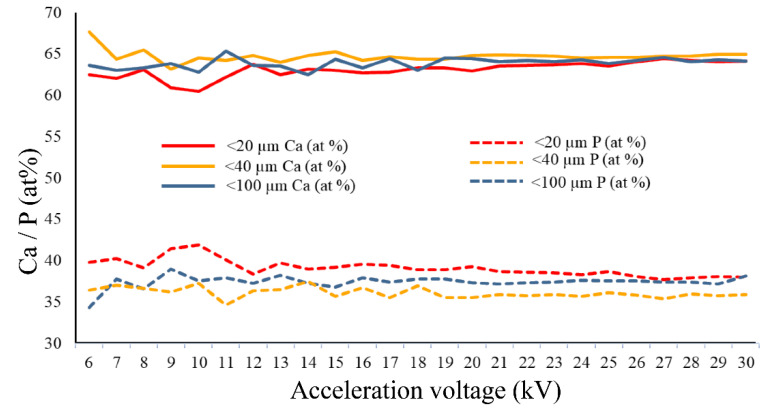
Variation of Ca and P concentration (at %) with the acceleration voltage and the particle size of CPs.

**Figure 5:**
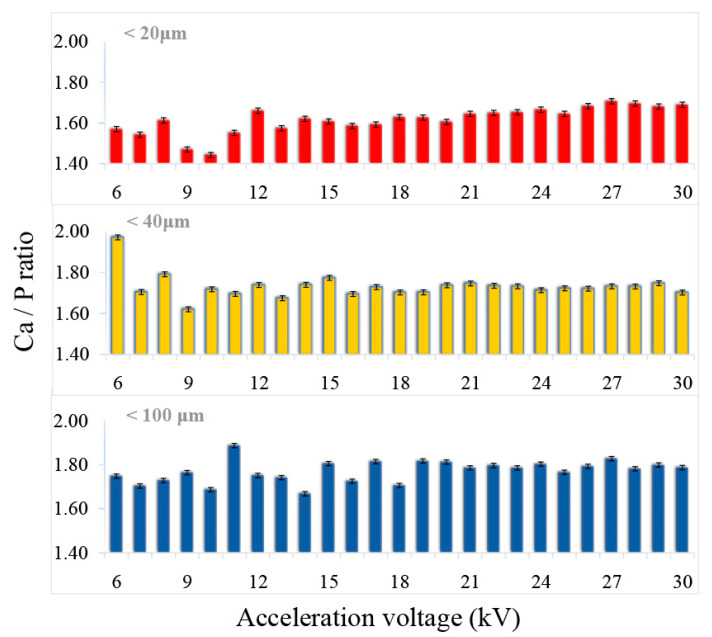
Variation of the Ca/P ratio with the acceleration voltage and the particle size of the CPs.

The compositional results corresponding to the Ca and P elements (Figure 4 and Figure 5) showed the uniformity of the signal intensity simultaneously with the increase of the acceleration voltage. This aspect can be attributed to the increase of the microvolume from which X-rays are generated (volume of interaction), thus, a larger number of atoms become generators of compositional signal, and the level of noise or background signal is reduced. For the both analysed elements, Ca and P, the variation of the signal with increasing voltage is the smallest for the samples with the particle size < 40 μm.

With the increase of the acceleration voltage (6–30 kV), the evolution in ascending trend of the Ca/P ratio was also observed. According to the literature [14], the typical acceleration voltage of a scanning electron microscope is between 0.1–30 kV. Thus, the higher the voltage, the greater the depth of emission of the X-ray signal from the sample. In the mainly used range by desktop instruments, 10–15 kV, very large variations were observed, these being reduced in the 15–20 kV voltage range. In the voltage range between 20 and 30 kV, the fluctuations had the lowest values.

According to previously published studies [24,40], in which complementary methods of compositional and structural characterisation (EDS, WDS, XRD and FT-IR) were used on these samples of non-stoichiometric hydroxyapatite, the Ca/P ratio has the value between 1.8 and 2 [25]. In the present study, the value closest to these results corresponds to the powder with particle size < 40 μm. A possible explanation consists in the presence within this particle size range of other particles from various dimensional ranges, the matrix factor being better attenuated. Therefore, the size of CPs particles that are compositionally characterised by the EDS method should not be very small (tending to the nanometric domain) nor too large, as additional X-ray reflections and also the fluorescence effect may occur. Apart from this, it was previously suggested that long terms exposures to the electron beam could possibly lead to the decomposition of the material, due to P-O covalent bonds breaking and P and O elements releasing and implicitly to slightly increased Ca/P values [9,38]. Moreover, the electron interaction volume with the sample is also influenced by the C and O reduction degree, resulting in elevated Ca and P proportions, causing probable errors in the microanalysis process [38]. Correlated with results in Figure 2, the detection of Ca and P differs function of signal intensity and acceleration voltage. Therefore, for an accurate determination of the Ca/P ratio, a corresponding excitation energy is required. Overall, the results demonstrate that, for particles with sizes lower than 40 μm, examined at acceleration voltage greater than 20 kV, the signal variation and the Ca/P values fluctuations are noticeably lower, with minimum estimated errors.

## 4. Conclusions

The EDS method is extensively used for X-ray microanalysis. This study highlighted the influence of the electron beam acceleration voltage and the particle size of the CPs particles on the EDS type microanalytical results. The variation of Ca and P concentrations is significant, up to an acceleration voltage of 20 kV, so a higher acceleration voltage value is required for the analysis of the elements in the CPs composition. The Ca/P ratio was calculated for each dimensionally sorted powder group, and the influence of particle size on the results was observed. In the 20–30 kV voltage range (recommended for CPs analysis), the smallest variations were observed in the compositional analysis of powders with dimensions smaller than 40 μm, which also contain particles with smaller dimensions (practically a mixture of particles with various sizes), thus, reducing the matrix factor and other effects that may alter the result. The results obtained in this paper confirm the need to adopt well-established working protocols for the characterisation of CPs powders with better accuracy and reproducibility of compositional results.

## Figures and Tables

**Table 1:** Average Ca/P ratio for CPs powders with different particle sizes. Standard deviation was calculated based on five measurements performed under repeatable conditions.

Particle Dimension	Average Ca/P Ratio	Standard Deviation
<20 µm	1.7128	0.0696
<40 µm	1.8344	0.0502
<100 µm	1.7720	0.0639
